
JOURNAL INFORMATION
==============================
NLM Title Abbreviation: CPT Pharmacometrics Syst Pharmacol
Journal ID: CPT Pharmacometrics Syst Pharmacol
NLM Journal ID: 101580011
Journal ID: PSP4

CPT: Pharmacometrics & Systems Pharmacology

EISSN: 2163-8306
Publisher: Wiley

ARTICLE INFORMATION
==============================
PMCID: PMC11474851
DOI: 10.1002/psp4.12826
Article ID: PSP412826
Article version: 1
Subjects: Issue Information, Issue Information

Issue Information

Electronic publication date: 2023 Nov 27
Publication date: 2023 Nov
Volume: 12
Issue: 11
Issue title: Dose Optimization in Oncology
First page: 1565
Last page: 1568
Copyright: © 2023 ASCPT
License: This is an open access article under the terms of the http://creativecommons.org/licenses/by/4.0/ License, which permits use, distribution and reproduction in any medium, provided the original work is properly cited.
License URL: https://creativecommons.org/licenses/by/4.0/

Figure count: 0
Table count: 0
Page count: 3

source-schema-version-number: 2.0
cover-date: November 2023
details-of-publishers-convertor: Converter:WILEY_ML3GV2_TO_JATSPMC version:6.4.9 mode:remove_FC converted:15.10.2024

==============================

An Official Journal of ASCPT and ISoP

### CPT: Pharmacometrics & Systems Pharmacology

Open Access

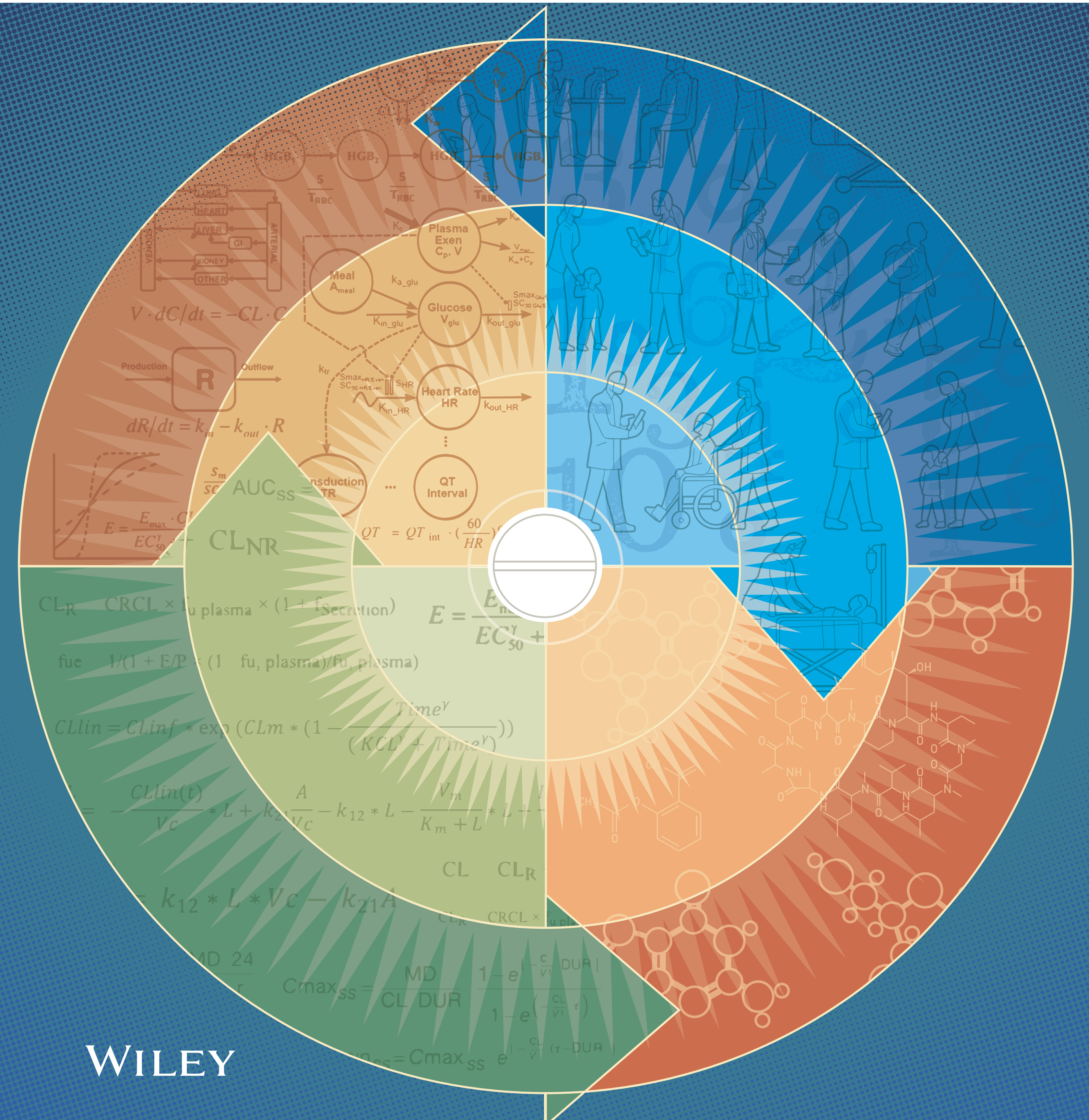

WILEY

### CPT: Pharmacometrics & Systems Pharmacology

WILEY

### Editor-in-Chief

France Mentré, MD, PhD, *School of Medicine of University Paris Diderot, Paris, France*

### Deputy Editor-in-Chief

Karen Rowland Yeo, PhD, *Certara UK Ltd., Sheffield, UK*

### Associate Editors

Paolo Denti, PhD, *University of Cape Town, Cape Town, South Africa*
Jonathan French, ScD, *Metrum Research Group, Tariffville, CT, USA*
Stefanie Hennig, MSc, PhD, *Certara Strategic Consulting, Brisbane, Australia*
Priya Jayachandran, PharmD, *Regeneron Pharmaceuticals, Inc., San Francisco, CA, USA*
Andrew Hooker, PhD, *Uppsala University, Uppsala, Sweden*
Sriram Krishnaswami, PhD, *Pfizer, Groton, CT, USA*
Ping Zhao, PhD, *Bill & Melinda Gates Foundation, Seattle, WA, USA*

### Editors-in-Training

Brian Cicali, PhD, *University of Florida, College of Pharmacy, Orlando, FL, USA*
Jane Knöchel, PhD, *AstraZeneca , Mölndal, Sweden*

### American Society for Clinical Pharmacology and Therapeutics

***Chief Executive Officer***
Abigail W. Gorman, *Alexandria, VA*
***Chief Operating Officer***
Elise S. Laffman-Johnson, *Alexandria, VA*
***Managing Editor***
Alethea B. Gerding, *Alexandria, VA*
***Deputy Managing Editor***
Alaina Webster, *Alexandria, VA*
***Senior Editorial Coordinator***
Aimee Greene, *Alexandria, VA*

### Editorial Board

Vivaswath S. Ayyar, PhD, *Janssen Research & Development, Spring House, PA*
Robert Bies, PharmD, PhD, *State University of New York at Buffalo, Buffalo, NY, USA*
Marc Birtwistle, PhD, *Clemson University Clemson, SC, USA*
Yuan Chen, PhD, *Genentech South San Francisco, CA, USA*
Marylore Chenel, PharmD, PhD, *Pharmetheus Uppsala, Sweden*
Emmanuel Chigutsa, PhD, *Eli Lilly and Company, Indianapolis, IN, USA*
Teresa Dalla Costa, PhD, *Federal University of Rio Grande do Sul - UFRGS Porto Alegre, Brazil*
Rajat Desikan, PhD, *GSK, UK, Middlesex, UK*
Narendra M Dixit, PhD, *Indian Institute of Science, Bengaluru, India*
Anne-Gaelle Dosne, PharmD, PhD, *Janssen Research & Development, Beerse, Belgium*
Thomas Dumortier, Eng, MSc, *Novartis Pharma AG, Basel, Switzerland*
James M. Gallo, PharmD, PhD, *University at Buffalo, Buffalo, NY, USA*
Wei Gao, PhD, *EMD Serono, Billerica, MA, USA*
Jérémie Guedj, PhD, *INSERM, Paris, France*
Manish Gupta, PhD, FCP, *GenMab; Princeton, NJ, USA*
Bart Hendriks, PhD, *Novartis Institutes for BioMedical Research Cambridge, MA, USA*
Nick Holford, MBChB, *The University of Auckland Auckland, New Zealand*
Brett A. Howell, PhD, *DILIsym Services Division, Simulations Plus, Inc., Research Triangle Park, NC, USA*
Manuel Ibarra, PhD, *Universidad de la República, Montevideo, Uruguay*
Siv Jönsson, PhD, *Uppsala University Sweden, Uppsala, Sweden*
Kristin Karlsson, PhD, *Uppsala University Uppsala, Sweden*
Helen Kastrissios, PhD, *QuanTx Consulting, Brisbane, Australia*
Ron Keizer, PhD, *InsightRX San Francisco, CA, USA*
Steven E. Kern, PhD, *Global Health Labs, Bellevue, WA, USA*
Akash Khandelwal PhD, *UCB Biosciences GmbH, Monheim am Rhein, Germany*
Andrzej Kierzek, PhD, *Certara QSP; Sheffield, United Kingdom*
Qi Liu, PhD, *US Food and Drug Administration, Silver Spring, MD*
James Lu, PhD, *Genentech, Inc., South San Francisco, CA, USA*
Rajanikanth (Raj) Madabushi, PhD, *US Food and Drug Administration, Silver Spring, MD*
Efthymios Manolis, MSc, *European Medicines Agency London, United Kingdom*
Samer Mouksassi, PharmD, PhD, *Certara, Cairo, Egypt*
Jackson K. Mukonzo, PhD, *Makerere University, Kampala Uganda*
Joakim Nyberg, PhD, *Pharmetheus, Uppsala, Sweden*
Elsje Pienaar, PhD, *Weldon School of Biomedical Engineering, Purdue University, West Lafayette, IN, USA*
Daniel Scholes Rosenbloom, PhD, *Merck & Co., Rahway, NJ, USA*
Joe Standing, PhD, *University College London London, United Kingdom*
Natasha Strydom, PhD, *Amgen, Inc., San Francisco, CA, USA*
Yuichi Sugiyama, PhD, *Riken Innovation Center Sugiyama Laboratory Yokohama, Japan*
Elin Svensson, PhD, *Uppsala University, Uppsala, Sweden*
Joel Tarning, PhD, *Mahidol-Oxford Tropical Medicine Research Unit Bangkok, Thailand*
Nadia Terranova, PhD, *Merck KGaA, Lausanne, Switzerland*
Michael Tortorici, PharmD, PhD, *Aro Biotherapeutics, Philadelphia, PA, USA*
Coen van Hasselt, PhD, *Leiden University, Leiden, the Netherlands*
Paolo Vicini, PhD, MBA, *Confo Therapeutics, Ghent, Belgium*
Camille Vong, PhD, *Novartis AG Pharma, Basel, Switzerland*
Xinyuan (Susie) Zhang, PhD, *Daiichi Sankyo, Inc. Basking Ridge, NJ, USA*

### Overview

*CPT: Pharmacometrics & Systems Pharmacology* (PSP) is an official open access journal of the American Society for Clinical Pharmacology and Therapeutics (ASCPT) and International Society of Pharmacometrics (ISoP).

*CPT: Pharmacometrics & Systems Pharmacology* is a cross-disciplinary journal devoted to publishing advances in quantitative (e.g., modeling and simulation) methods as applied in pharmacology, physiology and therapeutics in humans. The journal welcomes original research articles, reviews and tutorials that bridge the following areas: pharmacometrics, modeling and simulation as applied to the design and evaluation of clinical trials, systems pharmacology modeling, particularly with a mechanistic link to human (patho)physiology, disease modeling, "population" or mixed-effects pharmacokinetics and pharmacodynamics (PKPD) modeling, modeling and simulation to support translational research, physiologically-based pharmacokinetics (PBPK), model-based meta-analyses of clinical trials, mechanism-based pharmacokinetic-pharmacodynamic modeling, computational pharmacology, bioinformatics, comparative efficacy, effectiveness and cost-effectiveness. Systems pharmacology may involve the application of systems biology approaches to study drug activities, targets and effects. The discipline is often defined with reference to engineering and pharmacological principles as the quantitative analysis of the dynamic interactions between drugs and a biologic system that aims to understand the behavior of the system as a whole. The common focus will be on quantitative methods that improve our understanding of pharmacology and therapeutics in humans.

**Abstracting and Indexing**
- Embase (Elsevier)
- SCOPUS (Elsevier)
- MEDLINE/PubMed indexing

**Society Information**

The American Society for Clinical Pharmacology and Therapeutics (ASCPT) was founded in 1900, and consists of over 2,200 professionals whose primary interest is to advance the science and practice of clinical pharmacology and translational medicine for the therapeutic benefit of patients and society. ASCPT is the largest scientific and professional organization serving the disciplines of Clinical Pharmacology and Translational Medicine.

**All articles published in this journal are freely available to read, download, and share. Articles are published under the terms of the Creative Commons Attribution Non-Commercial License which permits use, distribution, and reproduction in any medium, provided the original work is properly cited and is not used for commercial purposes.**

**Disclaimer**

The Publisher, the American Society for Clinical Pharmacology and Therapeutics, the International Society of Pharmacometrics, and Editors cannot be held responsible for errors or any consequences arising from the use of information contained in this journal; the views and opinions expressed do not necessarily reflect those of the Publisher, the American Society for Clinical Pharmacology and Therapeutics, the International Society of Pharmacometrics or Editors, neither does the publication of advertisements constitute any endorsement by the Publisher, the American Society for Clinical Pharmacology and Therapeutics, the International Society of Pharmacometrics or Editors of the products advertised.

**Publisher**

*CPT: Pharmacometrics & Systems Pharmacology* is published by Wiley Periodicals, LLC, 101 Station Landing, Suite 300, Medford, MA 02155; Telephone: 781-388-8200; Fax: 781-388-8210.

CONTENTS

CPT: Pharmacometrics & Systems Pharmacology

### PERSPECTIVES

### EDITORIAL

1569 Role of pharmacometrics and systems pharmacology in facilitating efficient dose optimization in oncology
**Priya Jayachandran, Rajat Desikan, Sriram Krishnaswami and Stefanie Hennig**

### PERSPECTIVE

1573 US Food and Drug Administration embraces using innovation to identify optimized dosages for patients with cancer
**Stacy S. Shord, Hao Zhu, Jiang Liu, Atiqur Rahman, Brian Booth and Issam Zineh**

### REVIEWS

### REVIEWS

1577 Model-informed drug development of autologous CAR-T cell therapy: Strategies to optimize CAR-T cell exposure leveraging cell kinetic/dynamic modeling
**Anna M. Mc Laughlin, Peter A. Milligan, Cassian Yee and Martin Bergstrand**

1591 Skipping a pillar does not make for strong foundations: Pharmacokinetic-pharmacodynamic reasoning behind the shape of dose–response relationships in oncology
**James W. T. Yates and Hitesh B. Mistry**

### TUTORIAL

### TUTORIAL

1602 Building an adaptive dose simulation framework to aid dose and schedule selection
**Richard Hooijmaijers, Ridhi Parasrampuria, Eleonora Marostica, Geraldine Ferron-Brady, Teun M. Post and Sandra A. G. Visser**

### RESEARCH

### CASE REPORT

1619 PK/PD model-informed dose selection for oncology phase I expansion: Case study based on PF-06939999, a PRMT5 inhibitor
**Cen Guo, Kai H. Liao, Meng Li, I-Ming Wang, Naveed Shaik and Donghua Yin**

### ARTICLES

1626 Model-based prediction of effective target exposure for MEN1611 in combination with trastuzumab in HER2-positive advanced or metastatic breast cancer patients
**Elena M. Tosca, Elisa Borella, Chiara Piana, Salim Bouchene, Giuseppe Merlino, Alessio Fiascarelli, Paolo Mazzei and Paolo Magni**

1640 Mixed effects modeling of radiotherapy in combination with immune checkpoint blockade or inhibitors of the DNA damage response pathway
**David Hodson, Hitesh Mistry, Sofia Guzzetti, Michael Davies, Anna Staniszewska, Paul Farrington, Elaine Cadogan, James Yates, Leon Aarons and Kayode Ogungbenro**

1653 Sabatolimab (MBG453) model-informed drug development for dose selection in patients with myelodysplastic syndrome/acute myeloid leukemia and solid tumors
**Siyan Xu, Na Zhang, Mikael L. Rinne, Haiying Sun and Andrew M. Stein**

1666 Copanlisib population pharmacokinetics from phase I–III studies and exposure–response relationships in combination with rituximab
**Peter N. Morcos, Jonathan Moss, Rupert Austin, Florian Hiemeyer, Pier Luigi Zinzani, Vita Beckert, Lidia Mongay Soler, Barrett H. Childs and Dirk Garmann**

1687 Characterizing the exposure–response relationship of idecabtagene vicleucel in patients with relapsed/refractory multiple myeloma
**Jamie N. Connarn, Han Witjes, Marielle van Zutphen-van Geffen, Rik de Greef, Timothy B. Campbell, Kristen Hege, Simon Zhou and Manisha Lamba**

1698 Guiding model-driven combination dose selection using multi-objective synergy optimization
**Jana L. Gevertz and Irina Kareva**

1714 A prognostic baseline blood biomarker and tumor growth kinetics integrated model in paclitaxel/platinum treated advanced non-small cell lung cancer patients
**Francis Williams Ojara, Andrea Henrich, Nicolas Frances, Yomna M. Nassar, Wilhelm Huisinga, Niklas Hartung, Kimberly Geiger, Stefan Holdenrieder, Markus Joerger and Charlotte Kloft**

1726 Mechanistically modeling peripheral cytokine dynamics following bispecific dosing in solid tumors
**Jared Weddell**

1738 A multistate modeling and simulation framework to learn dose–response of oncology drugs: Application to bintrafusp alfa in non-small cell lung cancer
**Han Liu, Ana-Marija Milenković-Grišić, Sreenath M. Krishnan, Siv Jönsson, Lena E. Friberg, Pascal Girard, Karthik Venkatakrishnan, Yulia Vugmeyster, Akash Khandelwal and Mats O. Karlsson**

1751 Model-based meta-analysis of non-small cell lung cancer with standard of care PD-1 inhibitors and chemotherapy for early development decision making
**David C. Turner, Russ Wada, Helen Zhou, Xiaowei Wang, Rik de Greef, Chandni Valiathan, Lindsey Zhang, Nancy Zhang, Mita Kuchimanchi, Tai-Tsang Chen, Marc Ballas and Sandra A. G. Visser**

1764 Clinical decision support for chemotherapy-induced neutropenia using a hybrid pharmacodynamic/machine learning model
**Jasmine H. Hughes, Dominic M. H. Tong, Vanessa Burns, Bobby Daly, Pedram Razavi, Jaap J. Boelens, Srijib Goswami and Ron J. Keizer**

1777 How large must a dose-optimization trial be?
**David C. Norris**

1784 Population pharmacokinetic and exposure-response analyses of pemigatinib in patients with advanced solid tumors including cholangiocarcinoma
**Xiaohua Gong, Ayman Akil, Andre Ndi, Tao Ji, Xiang Liu, Mark Lovern and Xuejun Chen**

1795 Killing a fly with a sledgehammer: Atezolizumab exposure in real-world lung cancer patients
**Sophie Marolleau, Alice Mogenet, Clara Boeri, Mourad Hamimed, Joseph Ciccolini and Laurent Greillier**

1804 A model-based approach leveraging in vitro data to support dose selection from the outset: A framework for bispecific antibodies in immuno-oncology
**Javier Sánchez, Christina Claus, Rosmarie Albrecht, Brenda C. Gaillard, Joana Marinho, Christine McIntyre, Tamara Tanos, Axel Boehnke, Lena E. Friberg, Siv Jönsson and Nicolas Frances**
